# Unveiling an odd fate after death: The isolated Eneolithic cranium discovered in the Marcel Loubens Cave (Bologna, Northern Italy)

**DOI:** 10.1371/journal.pone.0247306

**Published:** 2021-03-03

**Authors:** Maria Giovanna Belcastro, Teresa Nicolosi, Rita Sorrentino, Valentina Mariotti, Annalisa Pietrobelli, Matteo Bettuzzi, Maria Pia Morigi, Stefano Benazzi, Sahra Talamo, Monica Miari, Nevio Preti, Lucia Castagna, Luca Pisani, Luca Grandi, Pietro Baraldi, Paolo Zannini, Daniele Scarponi, Jo De Waele

**Affiliations:** 1 Department of Biological, Geological and Environmental Sciences, Alma Mater Studiorum-University of Bologna, Bologna, Italy; 2 Department for the Cultural Heritage, Alma Mater Studiorum-University of Bologna, Ravenna, Italy; 3 Department of Physics and Astronomy, Alma Mater Studiorum-University of Bologna, Bologna, Italy; 4 Department of Chemistry “Giacomo Ciamician”, Alma Mater Studiorum-University of Bologna, Bologna, Italy; 5 Superintendency of Archaeology, Fine Arts and Landscape of the provinces of Bologna, Modena, Reggio Emilia and Ferrara, Bologna, Italy; 6 Gruppo Speleologico Bolognese (GBS)—Unione Speleologica Bolognese (USB), Bologna, Italy; 7 Department of Chemical and Geological Sciences, University of the Studies of Modena and Reggio Emilia, Modena, Italy; University at Buffalo - The State University of New York, UNITED STATES

## Abstract

An isolated human cranium, dated to the early Eneolithic period, was discovered in 2015 at the top of a vertical shaft in the natural Marcel Loubens gypsum Cave (Bologna area, northern Italy). No other anthropological or archaeological remains were found inside the cave. In other caves of the same area anthropic and funerary use are attested from prehistory to more recent periods. We focused on investigating the circumstances surrounding the death of this individual, since the cranium shows signs of some lesions that appear to be the results of a perimortem manipulation probably carried out to remove soft tissues. Anthropological analyses revealed that the cranium belonged to a young woman. We analysed the taphonomic features and geological context to understand how and why the cranium ended up (accidentally or intentionally) in the cave. The analyses of both the sediments accumulated inside the cranium and the incrustations and pigmentation covering its outer surface suggested that it fell into the cave, drawn by a flow of water and mud, likely from the edges of a doline. The accidental nature of the event is also seemingly confirmed by some post-mortem lesions on the cranium. The comparison with other Eneolithic archaeological sites in northern Italy made it possible to interpret the find as likely being from a funerary or ritual context, in which corpse dismemberment (in particular the displacement of crania) was practiced.

## Introduction

An isolated human cranium (skull without mandible) was discovered in 2015 during the exploration of new branches of a natural gypsum cave near Bologna (Northern Italy). The cave–named ‘Marcel Loubens’ after a French speleologist who died in 1952 at the age of 29 in an accident in Pierre Saint Martin Cave (France) during an exploration–is situated in the central area of the ‘Dolina dell’Inferno’ (Hell’s sinkhole), the largest karst depression (900 x 600 m, 125 m deep) in the ‘Parco Regionale dei Gessi Bolognesi e Calanchi dell’Abbadessa’ (San Lazzaro di Savena, Bologna, Emilia Romagna, Italy) [1].

**Fig 1:**
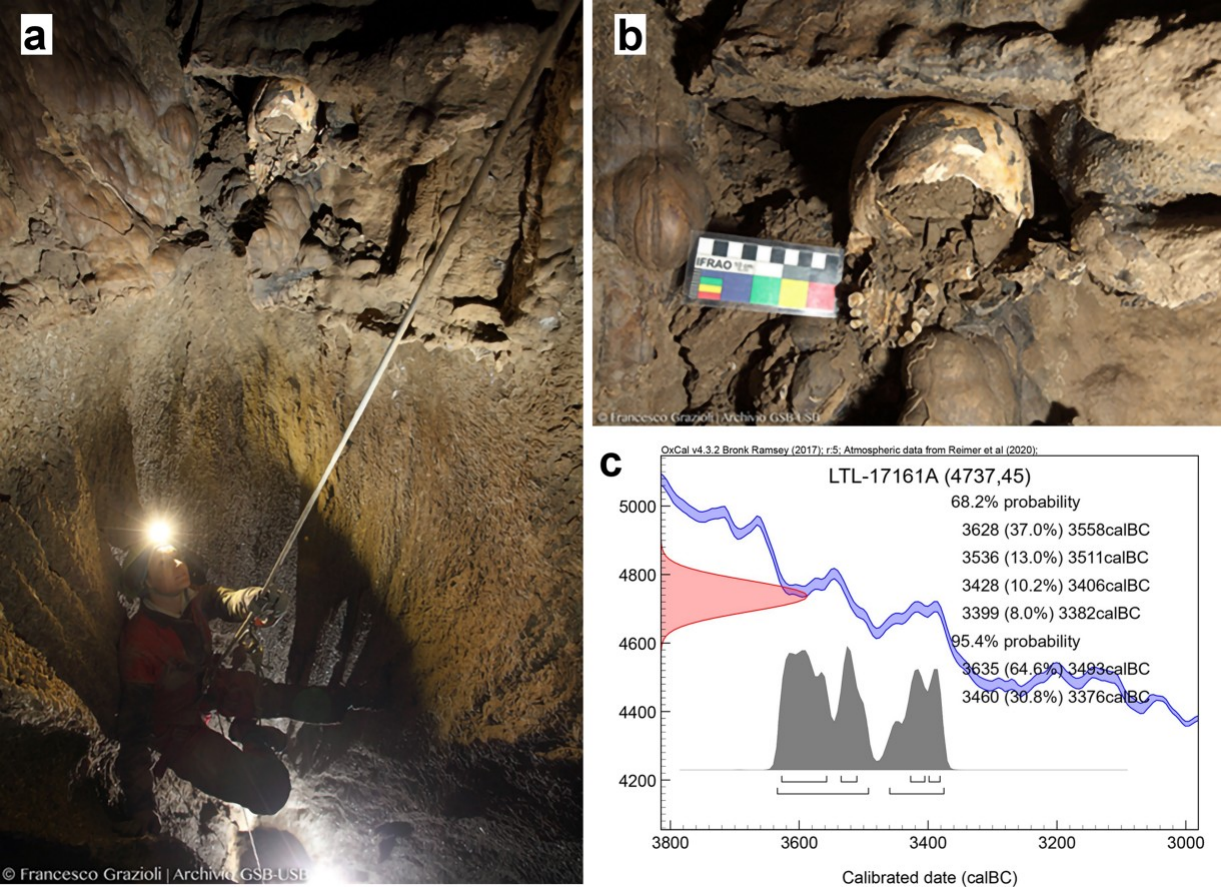
MLC on the top of the shaft and Lucia Castagna (a), the young archaeologist of GSB-USB that secured and recovered the cranium (Archive SABAP-BO/GSB-USB, ph. F. Grazioli; [The individual in this manuscript has given written informed consent (as outlined in PLOS consent form) to publish these case details]). Detail of MLC recovery (b) (Archive SABAP-BO/GSB-USB, ph. F. Grazioli). Calibrated conventional radiocarbon dates of LTL17161A sample (c) by CEDAD (‘Centro di Datazione e Diagnostica dell’Università del Salento’).

The cranium (hereafter referred to as the “MLC”–Marcel Loubens cranium) was found at the top of a shaft, which was reached by an artificial 12-metre technical climb from the lower lying ‘Meandro della cattiveria’ (Maze of Malice) (thus named because of the difficulties in moving through it). It was at a depth of 26 m below the ground level, in a cave that is mostly the result of a small temporary sinking creek, currently without any geomorphological evidence at the ground level (Fig 1A and 1B). The Marcel Loubens Cave is one of the sinking points likely connected with the nearby ‘Coralupi-Pelagalli’ karst system, whose waters are directed westward to the Zena River valley [2,3].

The cranium was vertically placed, exposing the basilar view: the cranial basis, palate, and occipital bone were visible and were embedded in a loamy matrix. Due to the difficulty in accessing the place where the cranium was found–with several passages that are not wide enough to allow more than one person to squeeze through–the recovery of the cranium was possible only on 7^th^ June 2017 thanks to the team of speleologists of the GSB-USB Bologna Caving Club. No other anthropological or archaeological remains were found [4].

The MLC was brought to the Laboratory of Physical Anthropology of the University of Bologna to be subjected to an anthropological study. A tooth (LM^3^) was used for the ^14^ C analysis at the CEDAD (‘Centro di Datazione e Diagnostica dell’Università del Salento’ [Dating and Diagnostic Centre of Salento University]). The Radiocarbon date (BP) is 4737 ± 45 [(δ^13^C (‰): -15.5 ± 0.3]. The ^14^C date was calibrated using the new calibration curve IntCal20 [5] range between 3,630 and 3,380 BC (with 68.2% probability) (Fig 1C), placing the MLC within the early Eneolithic phase in Northern Italy [6,7], even though for this area there is a general agreement in considering the 3600 BC as the beginning of the Eneolithic. In this area, a coeval anthropic frequentation was already documented in the Farneto Cave and the nearby deposit called ‘Sottoroccia del Farneto’ (Farneto rock shelter), just 600 metres away from the Marcel Loubens Cave. There, from 1924 to 1969, the explorer Luigi Fantini discovered some artefacts and multiple fragmented human remains, belonging to more than 25 individuals [8–10]. The site has been attributed to the Early Eneolithic period [11–13] and new, recently performed dating seems to confirm this chronology (data unpublished). The use of natural cavities for funerary purposes is known to have been common in the Emilia Romagna region for a long period, during the first half of the 3^rd^ millennium BC–as the radiocarbon dates of the ‘Tana della Mussina’ Cave [14] and the analysis of grave goods of the ‘Re Tiberio’ Cave [15] have shown–up to the 2^nd^ millennium BC, as suggested by Early Bronze Age finds [7,16].

The archaeological framework, the discovery of an isolated cranium, and the absence of any other archaeological and anthropological evidence in the Marcel Loubens cave give rise to many questions as to the interpretation of the context. Chronological data set the find within a broad funerary framework characterized by a focus on the crania [17–19]. Thus, the first aim of this study was to assess whether the cranium had undergone particular treatments. The next step was to determine if the cranium ended up in the cave accidentally or if it was placed there intentionally. Therefore, anthropological and taphonomical analyses were carried out taking the geological context into account.

## Materials and methods

All necessary permits were obtained for the described study by ‘Soprintendenza Archeologia, Belle Arti e Paesaggio per la città metropolitana di Bologna e le province di Modena, Ferrara e Reggio Emilia’. Access to the studied specimen (MLC-1) was granted by M.M., referent for the above-mentioned Institution, which complied with all relevant regulations and is the only beneficiary of concessions for excavation, study of materials, publication of results. Further study permits for this specimen can be evaluated upon request.

After the discovery and before removing the sediments filling the cranium, the MLC was subjected to a Computed Tomography (CT) scan in order to uncover any information regarding its original status at the time of its arrival at the Laboratory. The scan was performed using a tomographic system developed at the Department of Physics and Astronomy of the University of Bologna. The system is equipped with a 200 kVp X-ray tube and a CCD-based detector with an Apogee Alta U32 camera (2184 × 1472 pixels), optically coupled to a structured CsI (Tl) scintillator screen [20]. Acquisition parameters are listed in Table 1. The X-ray tube’s integrated aluminium (Al) filter (1 mm thickness) was coupled to an additional iron (Fe) beam filter (3 mm thickness) in order to increase the average energy of the beam and reduce the beam-hardening artefacts in tomographic images [S1 Video]. The tomographic reconstruction was obtained using an in-house software application based on a standard Filtered Back-Projection algorithm [21], while VG Studio Max software was used for the 3D rendering of the CT data. Later, the sediments were removed and analysed to understand the type, place, and possibly time of their formation.

**Table 1:** Acquisition parameters of the two CT scans carried out on the MLC.

	Scan 1	Scan 2
Tube voltage	200 kV	130 kV
Tube current	3.7 mA	140 μA
Beam filtration	3 mm Fe	no
Exposure time/frame rate	6 s	5 fps
Frame average	1	4
Number of projections	900	900
Angular range	360^o^	360^o^
Detector pixel size	264 μm	127 μm
Magnification	1.295	1.24
Voxel size	204 μm	102.4 μm

Two samples of sediment (~72.1 g and 67.8 g dry weight each) were randomly selected and soaked for 8 hours in a hydrogen peroxide solution (6% H_2_O_2_), filtered with a 63 μm-mesh sieve, and dried in an oven at 40°C. Then the sample residue was weighed, qualitatively observed under a binocular microscope, and described.

Afterwards, the cranium was reconstructed by repositioning its various fragments using only a surgical adhesive tape and modelling clay, both easy to remove. This permitted a full reconstruction enabling morphological and metrical observations and making possible a new session of CT scans for a better observation of the overall and inner structures, and to create a virtual record of the specimen. A new tomographic analysis was then performed in the same Department of Physics and Astronomy of the University of Bologna using a different CT system equipped with a microfocus X-ray source, working at a lower power but capable of providing better image quality. The main system components were a Varian PaxScan 2520D flat-panel detector (25 × 20 cm^2^, 1536 × 1920 pixels, 127 μm pixel size, 1–10 fps, 14 bits ADC) and a Kevex PXS-10 microfocus X-ray tube (130 kVp, 0.5 mA maximum current, 5 μm minimum focal spot size). The system was equipped with a two-axis translation stage for the detector, a vertical translation axis for the X-ray tube, and a micrometric rotary table for the object to be analysed [22]. Table 1 also shows the acquisition parameters for the second scan. It is worth noting that, in this case, the spatial resolution is higher (voxel size equal to half that of the first scan) [S2 and S3 Videos]. Moreover, four radiographic images were taken per angular step, and averaged in order to reduce noise and improve image quality.

To reconstruct the biological profile, we followed the customary anthropological standards [23]. In detail, the Acsádi and Nemeskéri index [24] was calculated for sex assessment. To achieve a better diagnosis, in the absence of the most dimorphic anatomical districts, a metric analysis was also conducted. A set of 20 measurements (S1 Table) according to Howells [25] were recorded and compared with those of a large sample (317: 164 males; 153 females) of modern European crania of known sex (Norway, Hungary, Austria; [25]) and 10 crania (5 males; 5 females) from modern identified human skeletons from the Certosa Cemetery of Bologna (Italy) (cf. [26]), as the latter are from the same geographical area as the MLC. A stepwise discriminant analysis was performed to determine the most dimorphic metric measurements across the known sample. Therefore, the stepwise selected measurements were analysed by leave-one-out cross-validation linear discriminant analysis (LDA), using final discriminant functions to predict the sex of the MLC. Finally, the MLC was projected into the Principal Component Analysis (PCA) previously computed on the known sample to explore its proximity with one sex or the other, as already done with other bones [27]. The cranial metric dataset was analysed in R v. 3.6.2 [28]. To estimate the age at death, the cranial suture closure method [29] was used. Occlusal dental wear [30,31] and the ossification of maxillary and palatine sutures [32] were also considered. Other biological features [non-metric traits (cf. [33]) and possible pathologies (cf. [34–41])] were recorded. Evidence of ante-, peri-, and postmortem injuries was studied, by examining possible bone modifications both macroscopically and under a stereomicroscope. To assess and distinguish the perimortem traumas (e.g. cut marks) due to intentional or accidental actions and natural events from postmortem ones, we used the criteria reported in the literature [42–48]. Bone fractures were also studied in order to distinguish those produced on the fresh bone (a smooth surface forming an acute or obtuse angle with the bone surface), dry bone (some organic components still present; intermediate features between fresh and mineralized fractures), or mineralized bone (a largely rough surface, mostly perpendicular to the bone surface) ([49–52]).

To better understand the chemical features of a red coloured area of the right frontal ectocranial surface, ED-XRF analysis (a non-invasive technique) was performed, using a Bruker ARTAX 200 instrument, Mo tube at 50kV and 0.7 mA, in Helium flux, focusing on an area of approximately 1 mm^2^. Raman microscopy, using a Labram from Jobin-Yvon with two lasers at 532 and 633 nm, was also performed. The instrument operated in backscattering with Edge filters. The dispersion was given by a monochromator coupled with a Charge Coupled Device detector having 256x 1024 pixels cooled to -70°C by the Peltier effect. Two fluorite lenses, with 50x and 100x power, made it possible to focus on a small area, thus avoiding any contact with the sample surface. The spectral resolution was 1 cm^-1^ and the possible spatial resolution was 1 micrometre. Spectra were recorded for long acquisition times, according to their intrinsic intensity.

## Results

### State of conservation

#### Anatomical parts preserved

**Fig 2:**
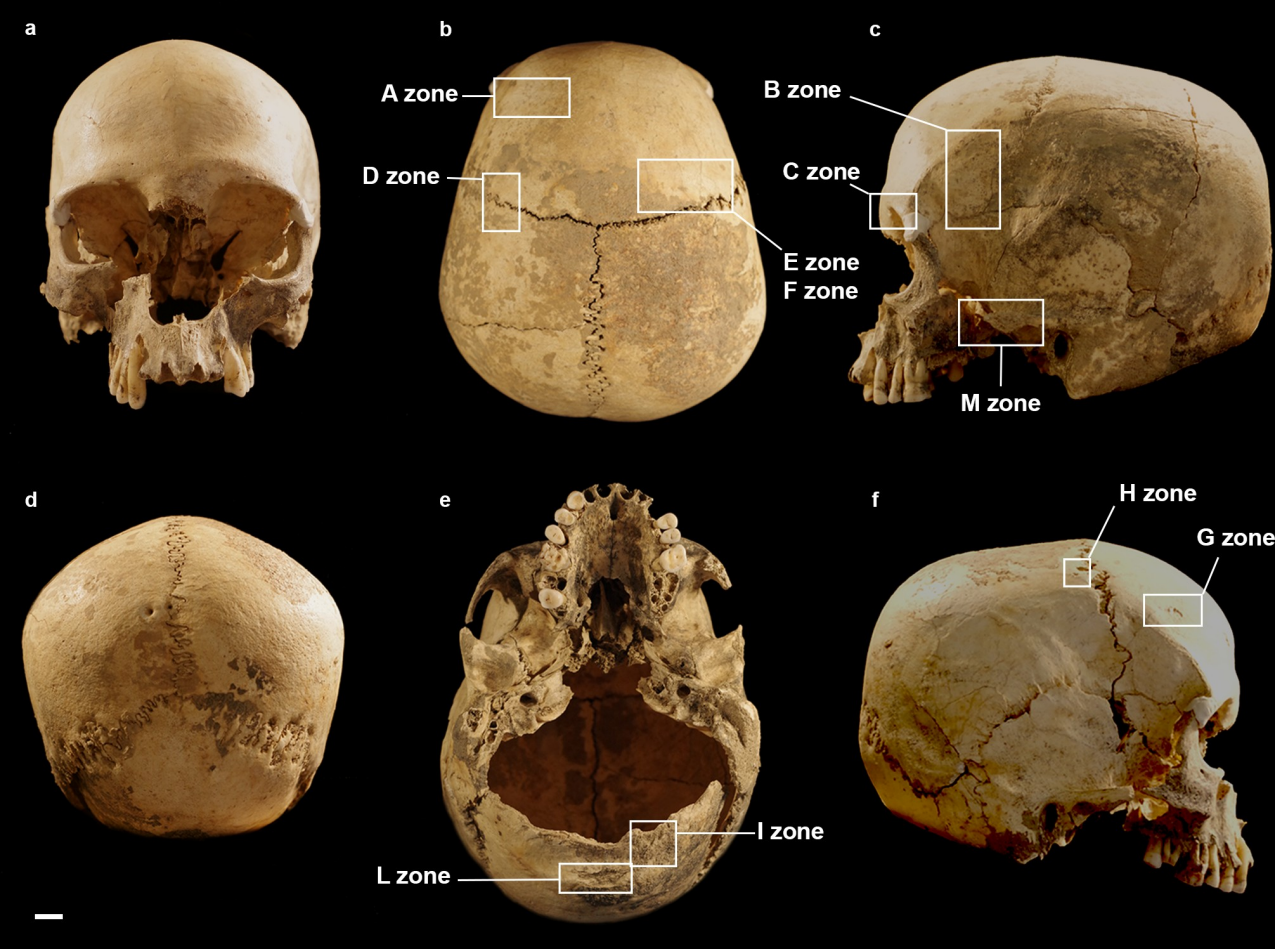
MLC in frontal (a), superior (b), left (c), posterior (d), inferior (e) and right (f) views. The boxes indicate the Zones (A-M) with the ectocranial lesions.

**Fig 3:**
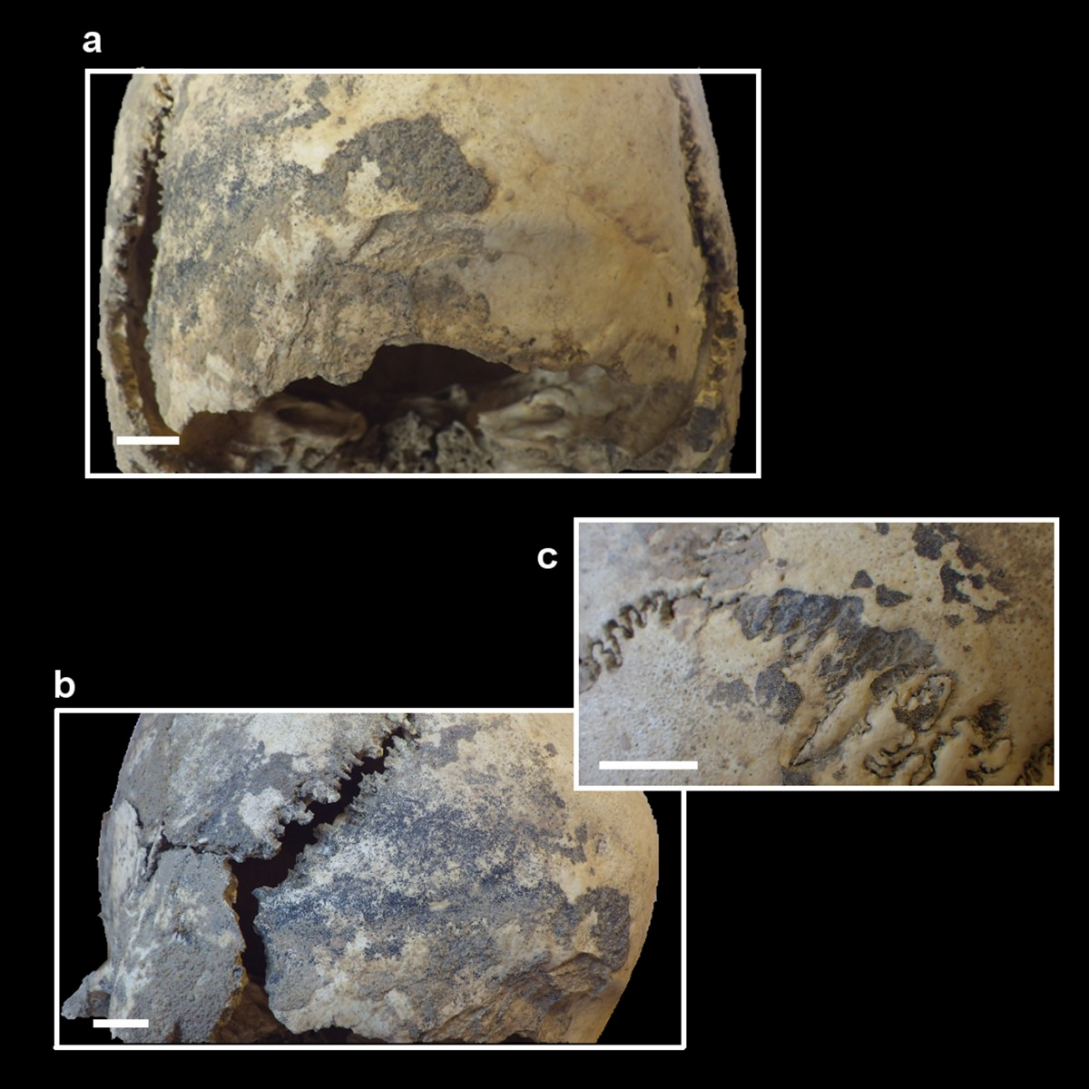
MLC—Detail of incrustations covering the occipital bone (a), left asteric region (b), and right lambdoid suture (c).

The cranium is in a rather good state of preservation (Fig 2). Its surface shows some radial and linear superficial cracks (affecting the frontal and parietal eminence). A full-thickness cross fracture affects the left parietal. Parietal and frontal bones are almost complete. The temporal bones lack a small portion of the squamous suture and the anterior portion of the zygomatic processes, while the left bone lacks the mastoid process. The occipital bone is only partially preserved. Most of the squama is present, while the *partes laterales* and *pars basilaris* are missing. The cranial base is not preserved and the large occipital fracture coinciding with the inferior nuchal line shows a curved, bevelled, almost regular profile. All edges and the preserved occipital surface are of the same colour and are irregularly covered by the same incrustations (Figs 2 and 3). A deformation is visible especially on the left side, making it impossible to join the occipital to the left parietal bone correctly (Figs 2E, 3A and 3B). The median area of the squama and the left part of the bone are partially covered by black-stained calcareous concretions and small black manganese oxide dots (Fig 3). The spheno-basilar synchondrosis is missing. The ethmoid bone is partially preserved. The splanchnocranium shows a great lacuna in the nasal region (nasal bones are absent). The zygomatic and maxillary bones are partially preserved. The palatine bones are broken; only small portions of their horizontal plates are preserved and joined to the maxillary bones. The alveolar processes and borders are not intact but all *alveoli* are visible (Fig 2). Some maxillary teeth are present (RC^1^, RP^3^, RP^4^, and RM^1^-M^3^ and LP^3^, LP^4^, LM^1^, LM3 –this last tooth was used for dating the specimen) and the others have been lost post-mortem.

#### Pigmentation and calcite crusts

The cranium is covered with a thin coating of a black material that does not react with H_2_O_2_, thus enabling us to rule out an organic staining (soot). These coatings consist of poorly crystalline manganese oxides and were formed by the flow of reduced water into the oxygenated cave environment. The reduced conditions are typical of water flowing through fine sediments and in the presence of large amounts of organic material (which became oxidized, thus consuming most of the O_2_ present in the water).

Calcite speleothems are common chemical deposits in gypsum caves in temperate regions. This is due to the common-ion effect: gypsum (CaSO_4_.2H_2_O) is much more soluble than calcite, so infiltration waters will rapidly dissolve gypsum and come close to saturation with this mineral. In the presence of CO_2_, this gas dissolves into the water and forms small amounts of HCO_3_^-^, which in the presence of Ca^2+^ (coming from the dissolved gypsum) rapidly combines to form calcite; the latter precipitates as flowstones. Calcite flowstones in gypsum caves generally form during periods when CO_2_ production in the soil is high, thus during warmer and wetter climates. Almost all carbonate speleothems found in the gypsum caves of the regions were formed during warm periods in the past [53,54].

**Fig 4:**
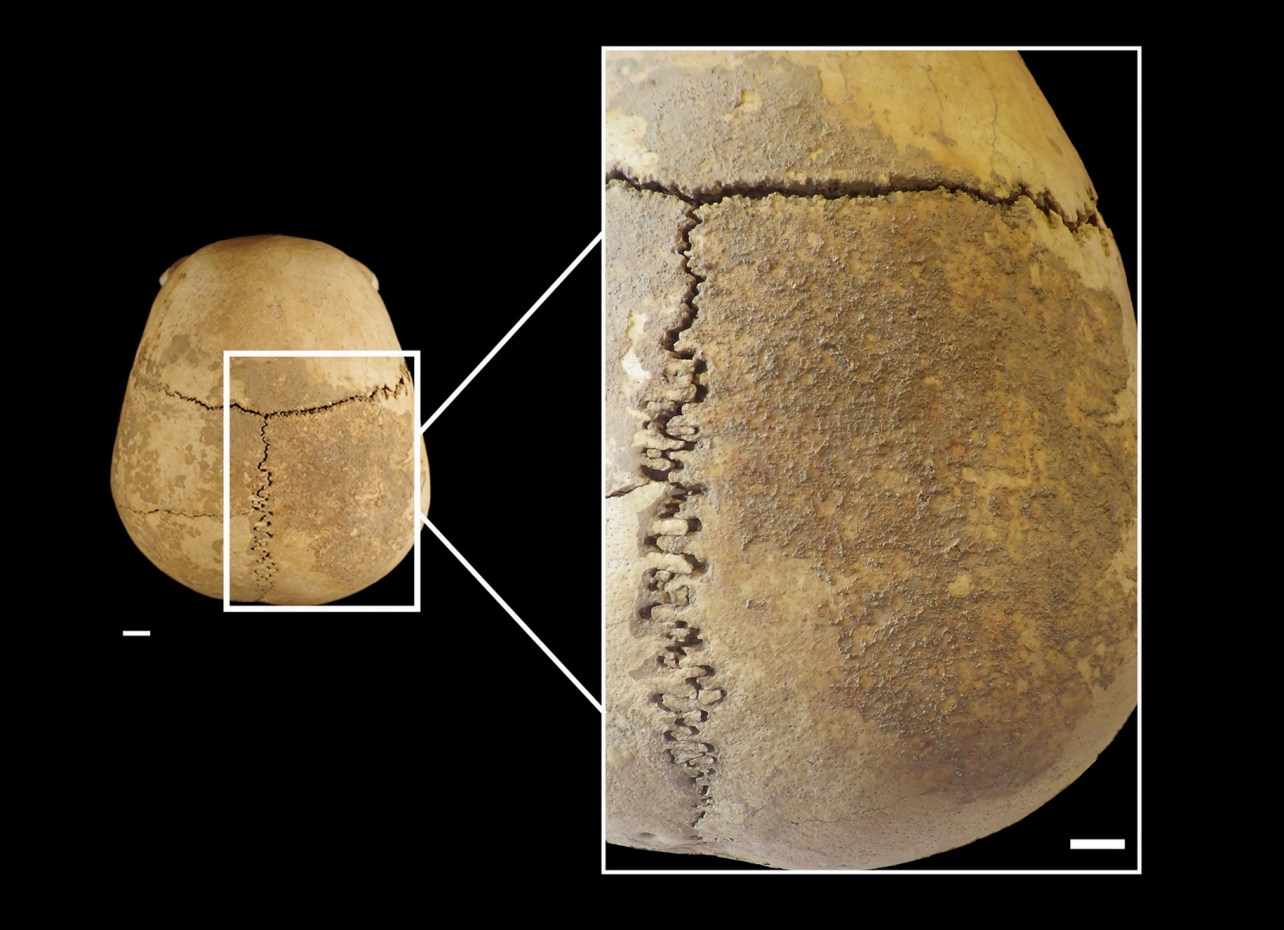
MLC—Detail of incrustations covering the right parietal bone and bregmatic area (superior view).

In detail, irregularly thick calcite crusts partially cover the external and the internal surface of the cranium, where clay fills holes, cavities, and the imprint of the vascular net. In particular, incrustations (Fig 4) cover the cranial vault, especially the bregmatic area. The right cranial wall is free of calcite crusts (except for the mastoid, where there is a darker accumulation than that described for the cranial vault), whereas the left wall shows a fine black pigmentation in direct contact with the cranium surface, later partially covered (except for the temporal squama) by lighter incrustations. The occipital squama is free of manganese oxides and calcite crusts, whereas the left part, i.e. the external eminence, is diffusely stained with manganese deposits and then covered by thick calcite crusts mixed with black pigments. The left asteric region and the mastoid process are partially covered by crusts. The right lambdoid suture is partly filled by well-preserved accumulations of manganese minerals, especially in the right asteric region (Fig 3C). Due to a cranial deformation, the left branch of this suture is not completely joined. The margins of the suture and the surrounding parietal, temporal, and occipital left surfaces are covered with a fine black Mn pigmentation. In the basilar view, the general fine manganese deposit stains the preserved parts, with the exception of the orbital cavities. The glenoid temporal fossae are stained by manganese pigments and the right one is covered by thick calcite at the level of the articular tubercle.

**Fig 5:**
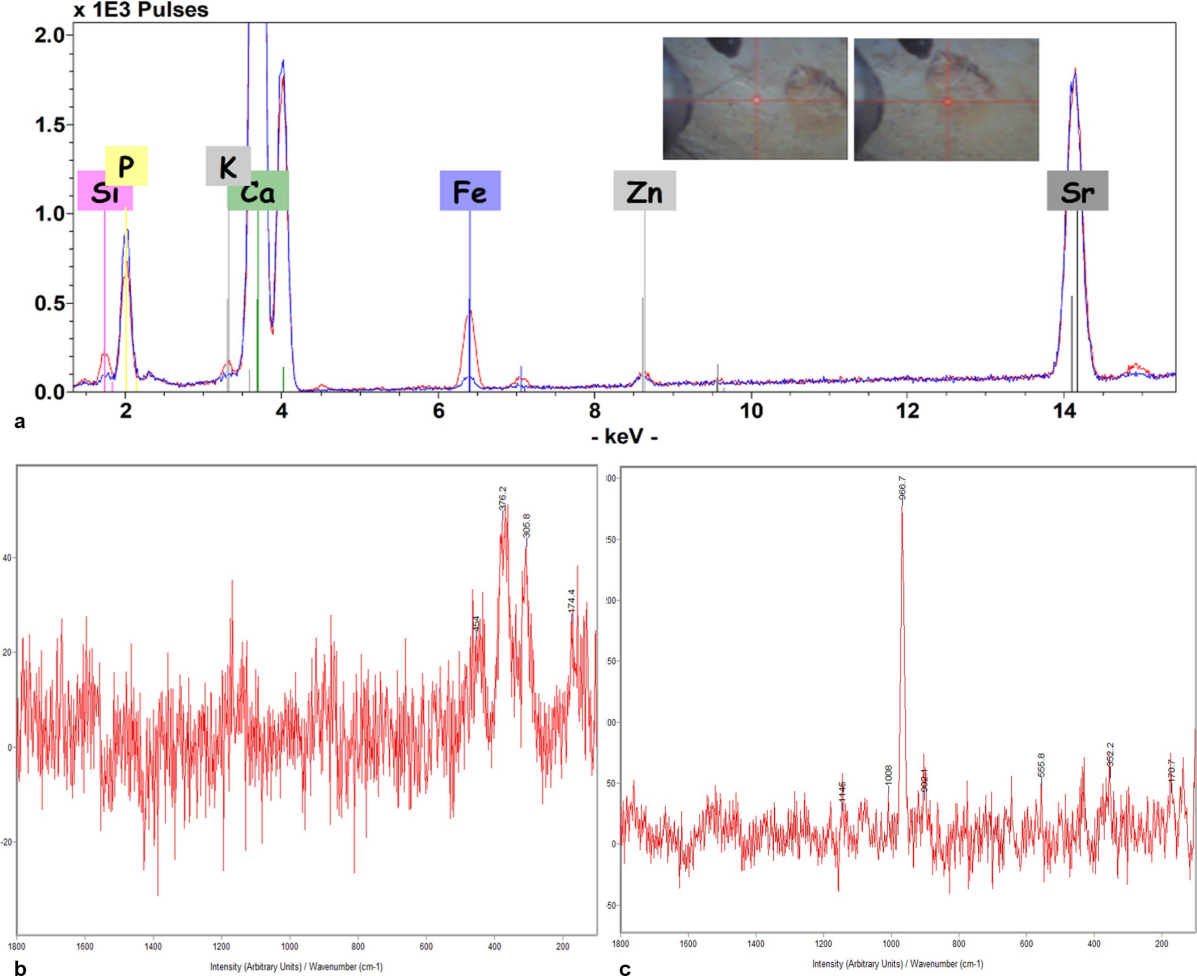
MLC—ED-XRF analysis on the G Zone: The blue and red lines refer to the area around (left picture) and inside (right picture) the lesion (a). Raman spectrum of the G Zone that shows the fluoro-apatite composition (b) and a yellow spot (Goethite) (c).

The ED-XRF and Raman analyses of the right frontal ectocranial surface showing a red pigmentation, where an ante mortem lesion has been found (see the result section), have revealed an increased amount of Fe, together with Si and K, being the amount of Ca, Sr and P, coming from the bone, substantially constant (Fig 5A).

**Fig 6:**
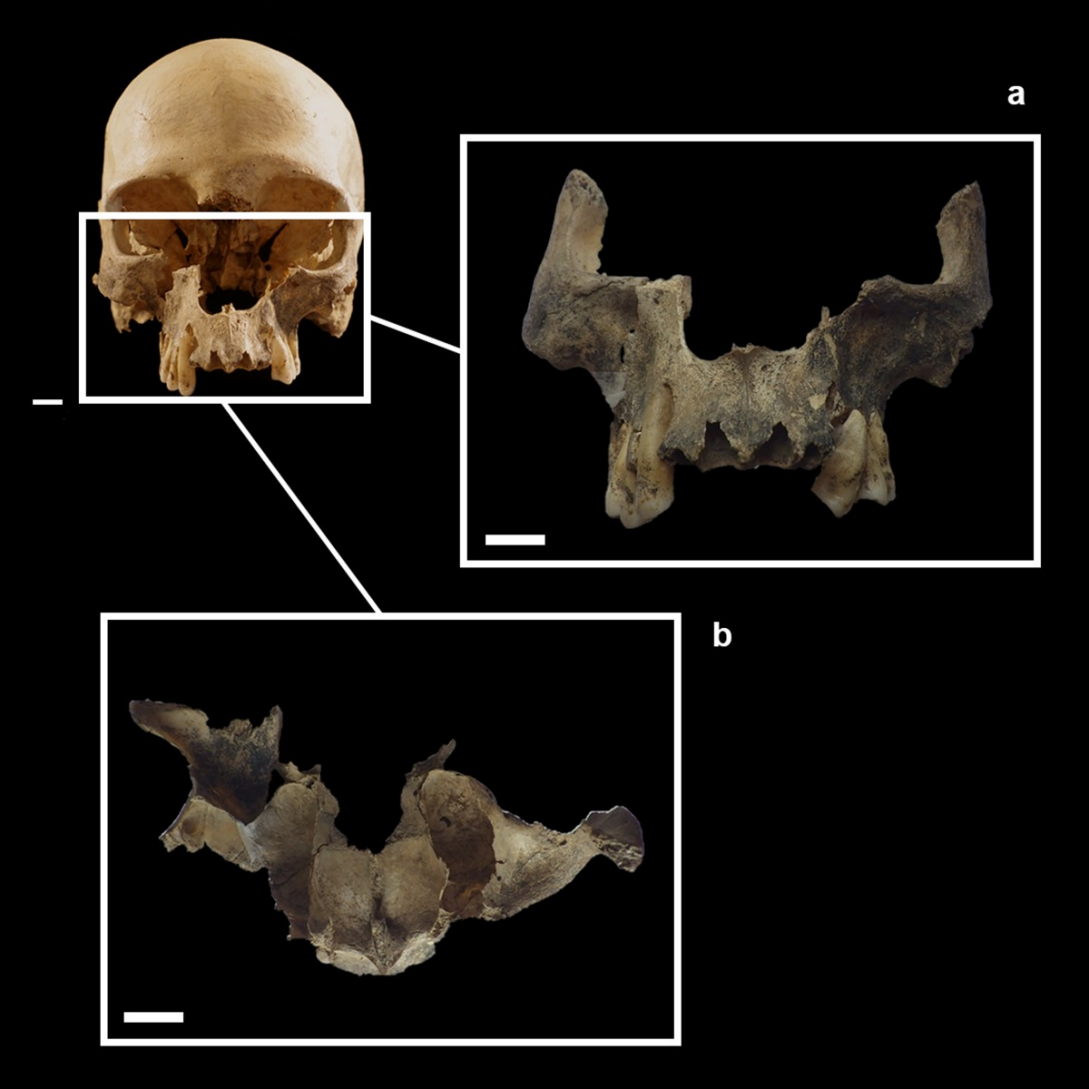
MLC–Cranium anterior view: Facial bones in the frontal (a) and superior (b) views.

A greyish-black rough crust also covers the margin of the cranial base fracture and other fractured margins. Finally, manganese pigments have stained the preserved facial bones on their external surface, also involving the palate and exposed teeth roots. The nasal floor and the preserved maxillary sinuses lack pigmentation and incrustations (Fig 6). A thin layer of clay covers the incrustations.

#### The sediments filling the cranial cavity

**Fig 7:**
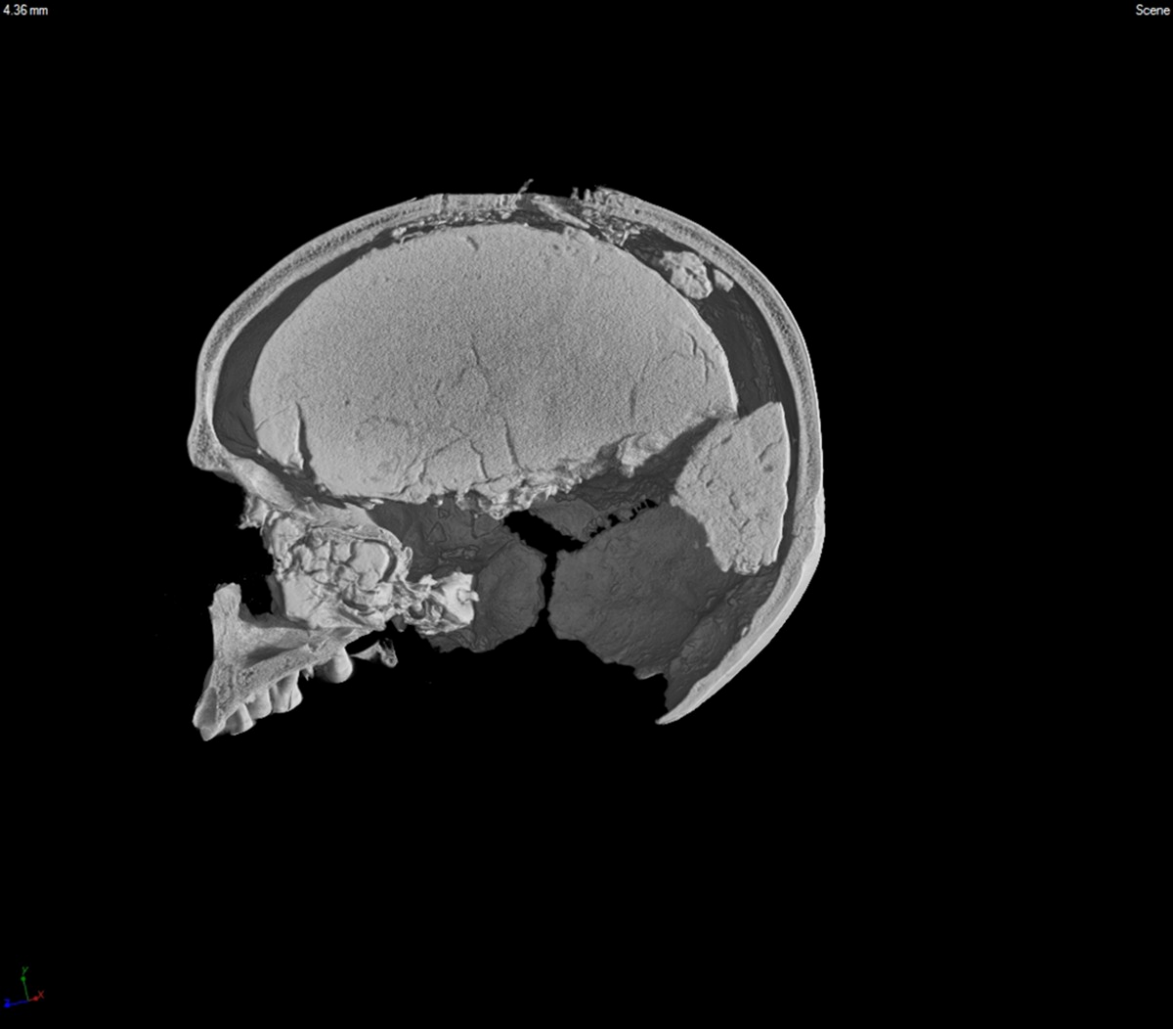
MLC—CT scan with sediment filling the endocranial cavity.

As for the sediment filling the cranium, this appears homogenous (Fig 7). The texture of both samples was sandy pelite with the coarser fraction (>0.63 mm) representing approximately 24% and 33% of each sample’s weight (16.1 g and 23.7 g, respectively). Worthy of note is the presence of sub-centimetre angular gravel-size particles, small fragments of gypsum, and several calcareous concretions (size <20 mm), along with the scattered presence of sub-millimetre-to-millimetre wood charcoal debris. Lastly, a few pulmonate and calciphile gastropods inhabiting elevations up to 2,600 m (*Vitrea* cf. *subrimata* and *Clausilia* sp.), along with other unidentifiable fragments of terrestrial mollusks, were found. No remains of micro- or meiofauna were seen in the residue.

### Biological profile

**Fig 8:**
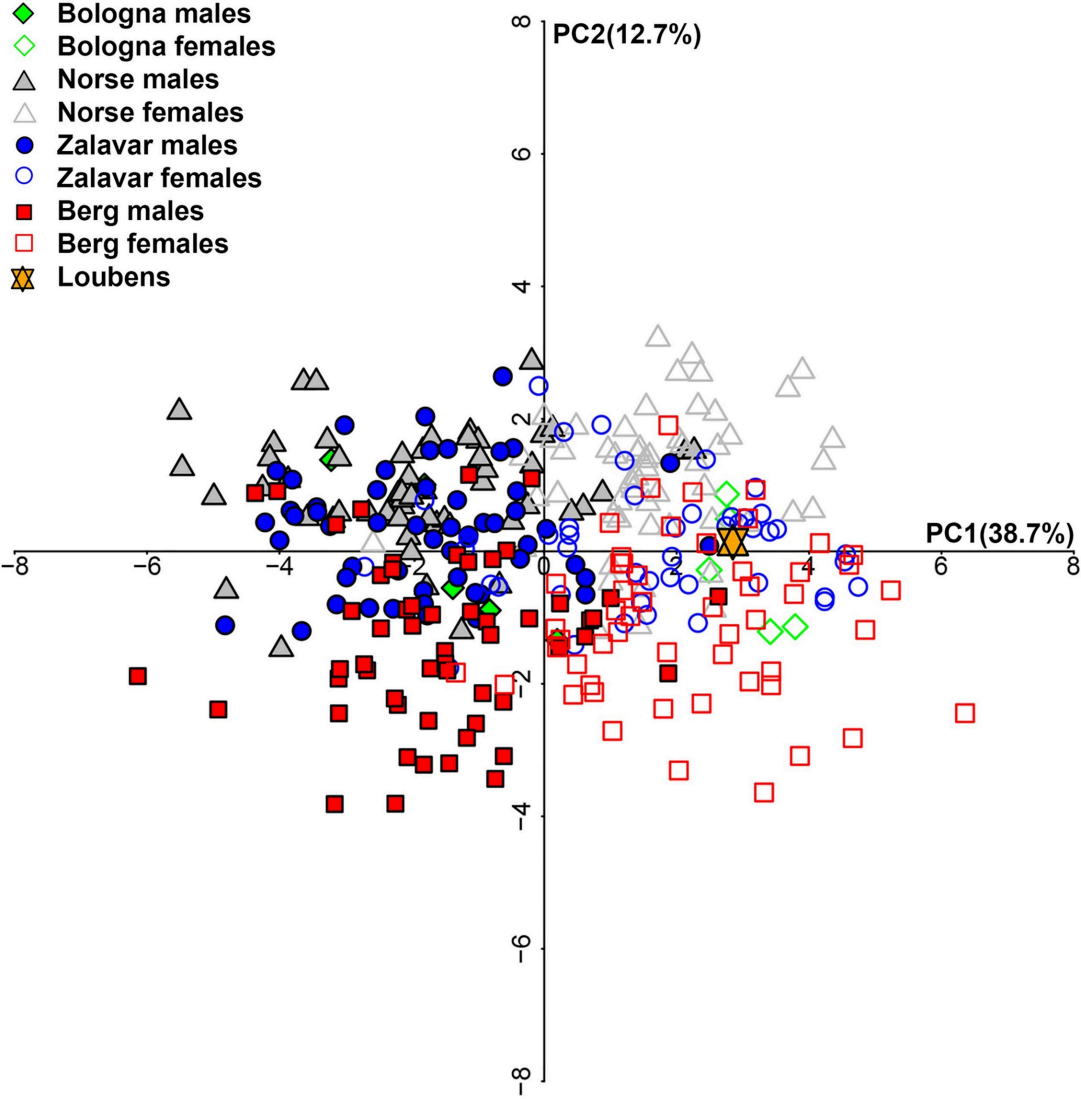
PCA plot showing that MLC falls within the area occupied by the European females.

The cranium falls within the young adult age bracket [23]. The external cranial suture obliteration indicated a wide age range (vault system: 18–45 yrs, lateral-anterior system: 25–49 yrs). The entire suture system is open on the outer and inner cranium surfaces. The age range was narrowed by observing the occlusal dental wear (24–35 yrs) and the maxillary-palatine suture ossification (26–35 yrs). The sexualization index (-0.55) indicates weak female traits. Stepwise discriminant analysis selected 15 out of 20 cranial linear measurements used in the analysis (S1 Table). Cross-validation LDA revealed that the 15 most dimorphic measurements (maximum cranial and nasion-occipital length, maximum frontal, biauricular, minimum cranial and biasterionic breadth, glabella projection, frontal chord, nasion-bregma subtense, parietal chord upper facial and nasal height, maxillo-alveolar breadth, mastoid height and breadth) separate males and females with 88.7% accuracy. The MLC ranks as female with a 99.6% posterior probability. Finally, the PCA plot (Fig 8) showed that the MLC falls within the area occupied by European females.

**Fig 9:**
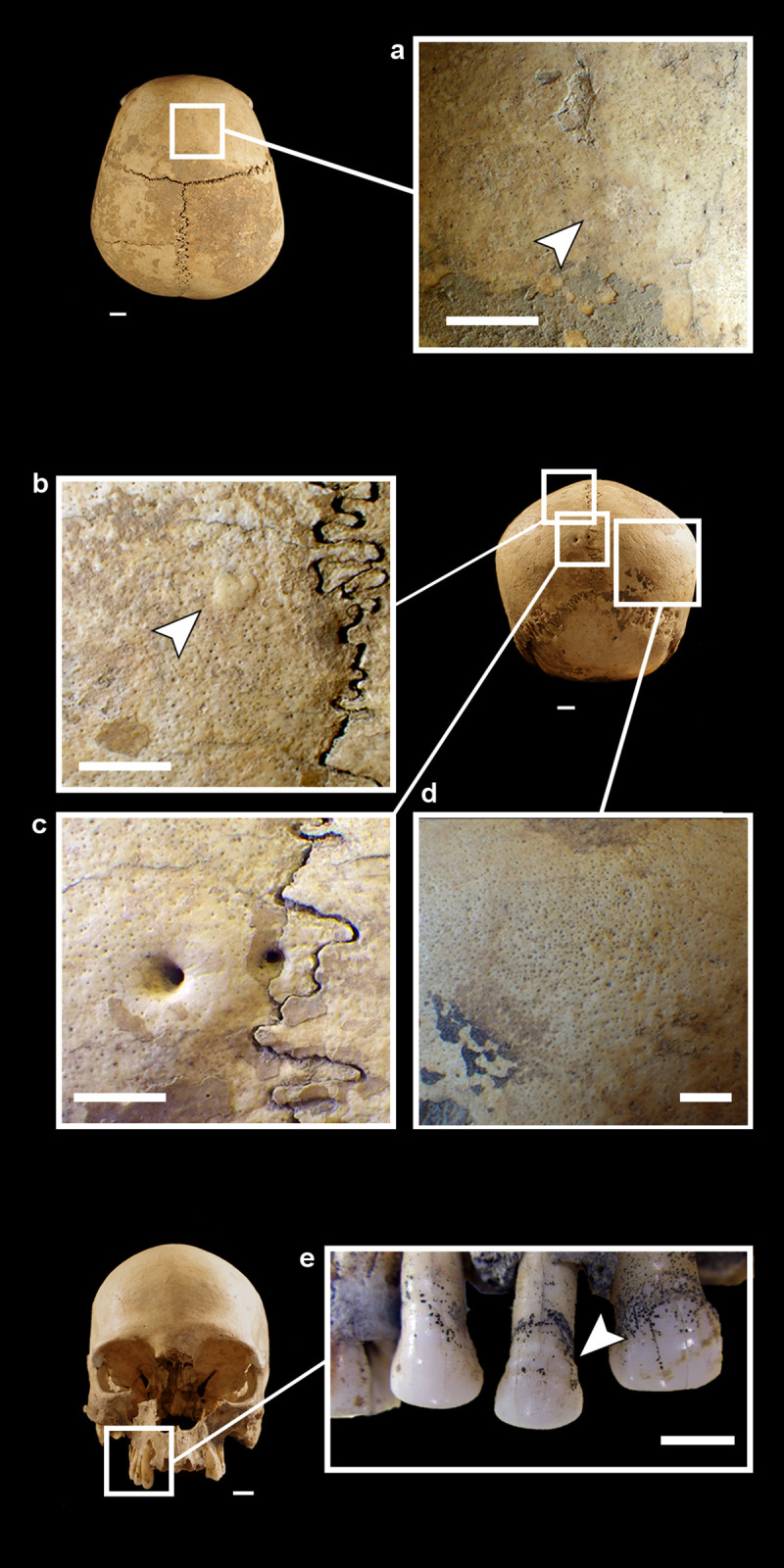
Details of button osteomas on the frontal (a) and left parietal (b) bones (about 4mm of diameter), double left parietal foramina (c), diffuse porosity (d), linear enamel hypoplasia (LEH) on LP^3^, RP^3^ and RP^4^ (e).

As regards some morphometric features, this cranium shows the vertical frontal bone and a flat posterior and lambdoid region. It shows a pentagon profile in the posterior view. The cranial index indicates brachycranic (81.8; length = 176 mm, breadth = 144 mm). Two lambdoid wormian bones, a weak trace of *os incae* due to the incomplete fusion of the transverse suture which separates the occipital squama (Fig 3), a double left parietal foramen (Fig 9C), and an accessory palatine suture on the right maxilla which delineates an anterior middle-palatine bone are detectable. Two small button osteomas (at the right frontal and left parietal) are present (Fig 9A and 9B). The external vault of the MLC shows a diffuse slight porosity (Fig 9D). Caries affects all molars (LM^1^, RM^1^, RM^2^, RM^3^, LM^3^) and slight deposits of calculus are present on both P^3^ and LM^1^. A medium degree of dental wear (4-5^th^ degree on the Smith’s scale) [34–36] and a linear enamel hypoplasia (LEH) on both P^3^ and RP^4^ can be seen (Fig 9E).

### Cranial lesions

**Fig 10:**
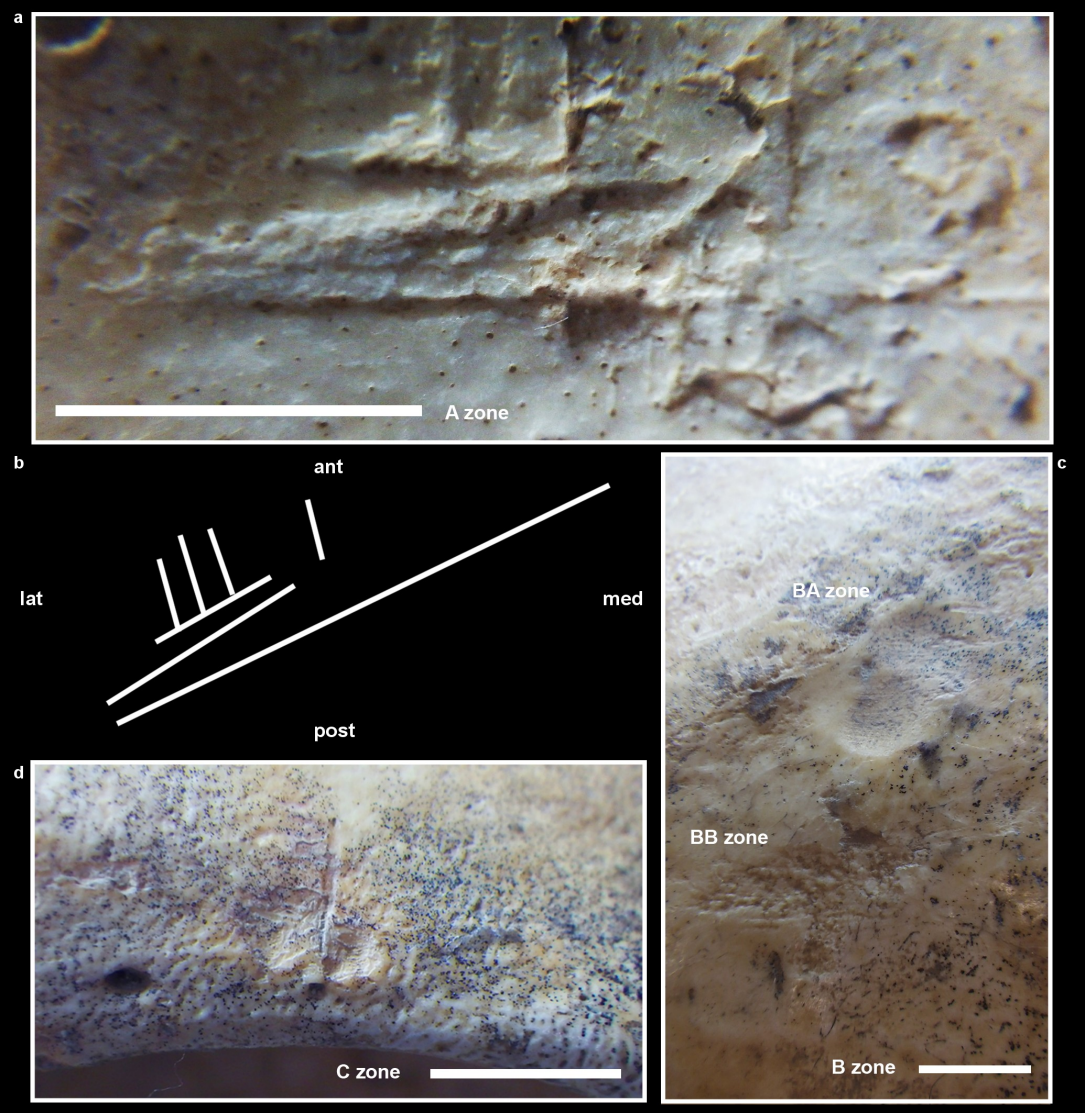
MLC- A Zone: detail of the grooves on left frontal squama (a) and drawing of the pattern (b); B Zone (c): Detail of the depressed and smooth (BA Zone) and scraped area (BB Zone) on the left portion of the frontal bone; C Zone (d): Detail of the detached area and groove on the left orbital margin.

**Fig 11:**
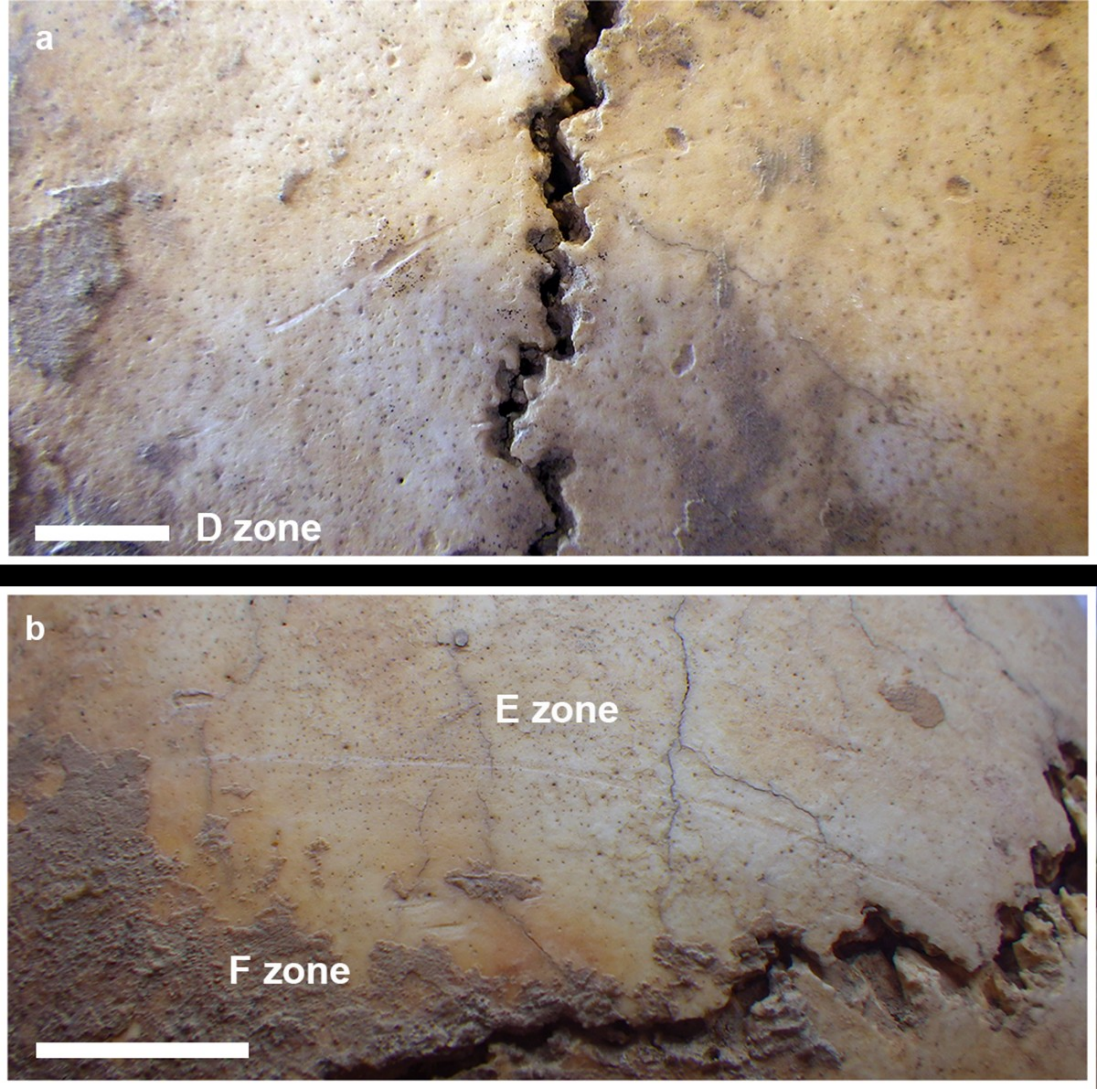
MLC—D Zone (a): Detail of the groove crossing the left coronal suture; E (linear groove) and F (three little marks) Zones on the right frontal squama (b).

**Fig 12:**
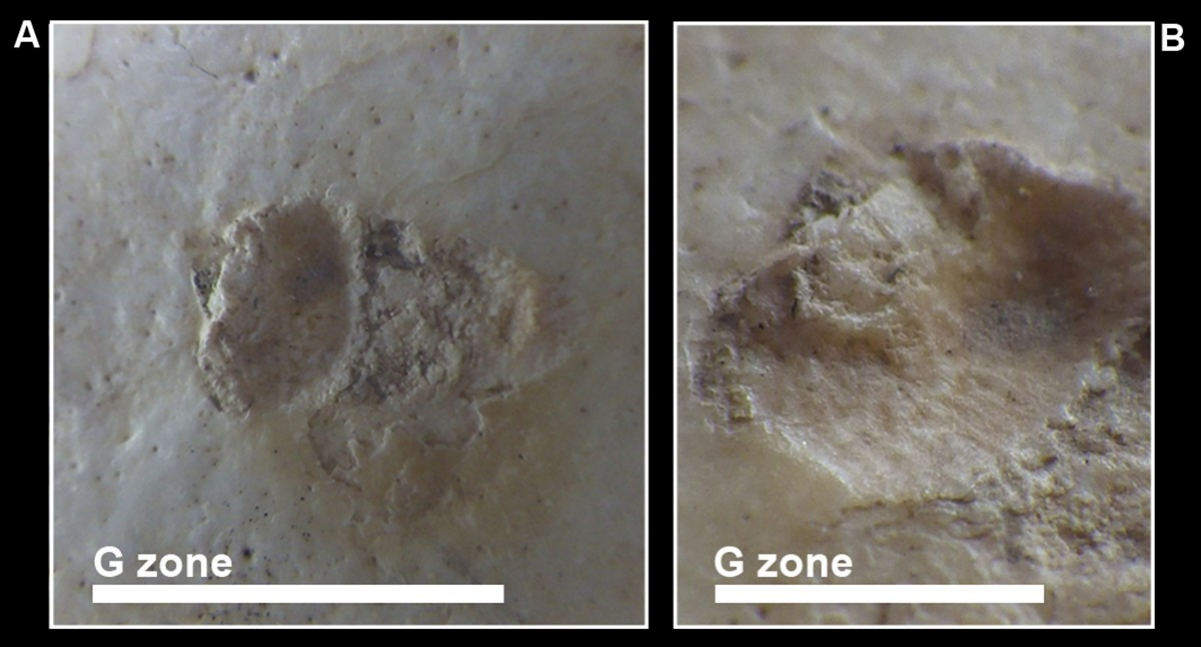
MLC- G Zone (a) on the right frontal squama. Note the red colour that stains the area; detail of the posterior part of the lesion: Microstriae in the bottom and bone remodelling in the posterior border are visible (b).

**Fig 13:**
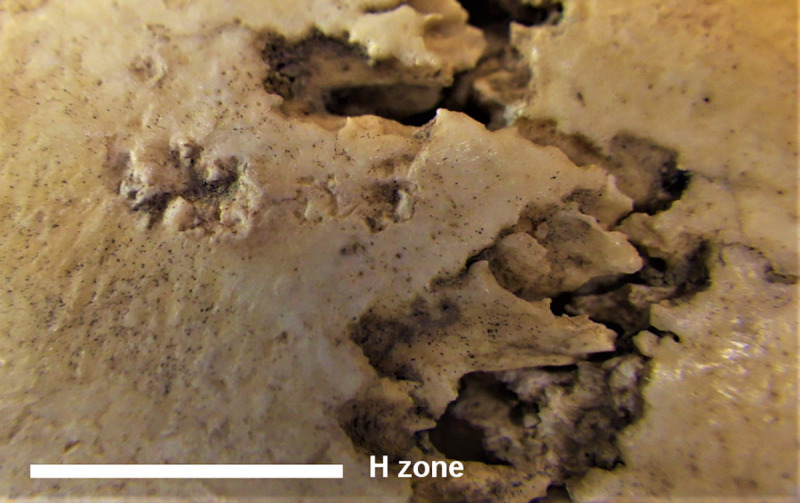
MLC—H Zone on the right parietal with detail of the areas with irregular pits.

**Fig 14:**
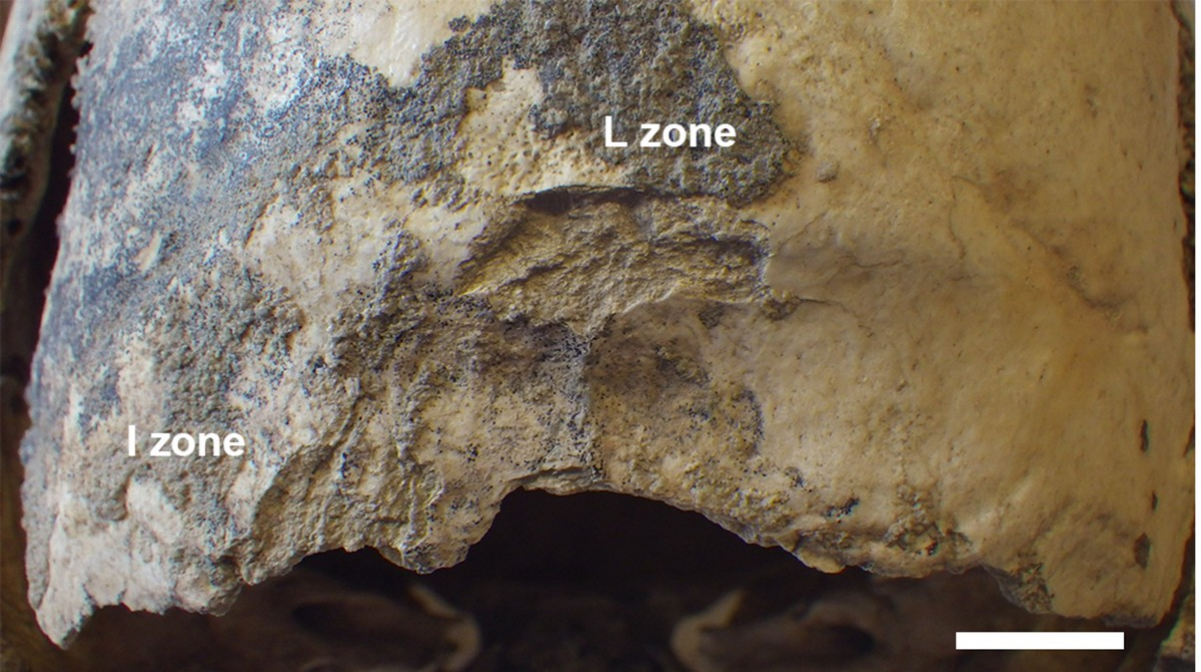
MLC—Occipital bone: Note the profile and margin of the fracture, the triangular-shaped and peeled area (I Zone) and the irregular and peeled surface on the inion (L Zone).

**Fig 15:**
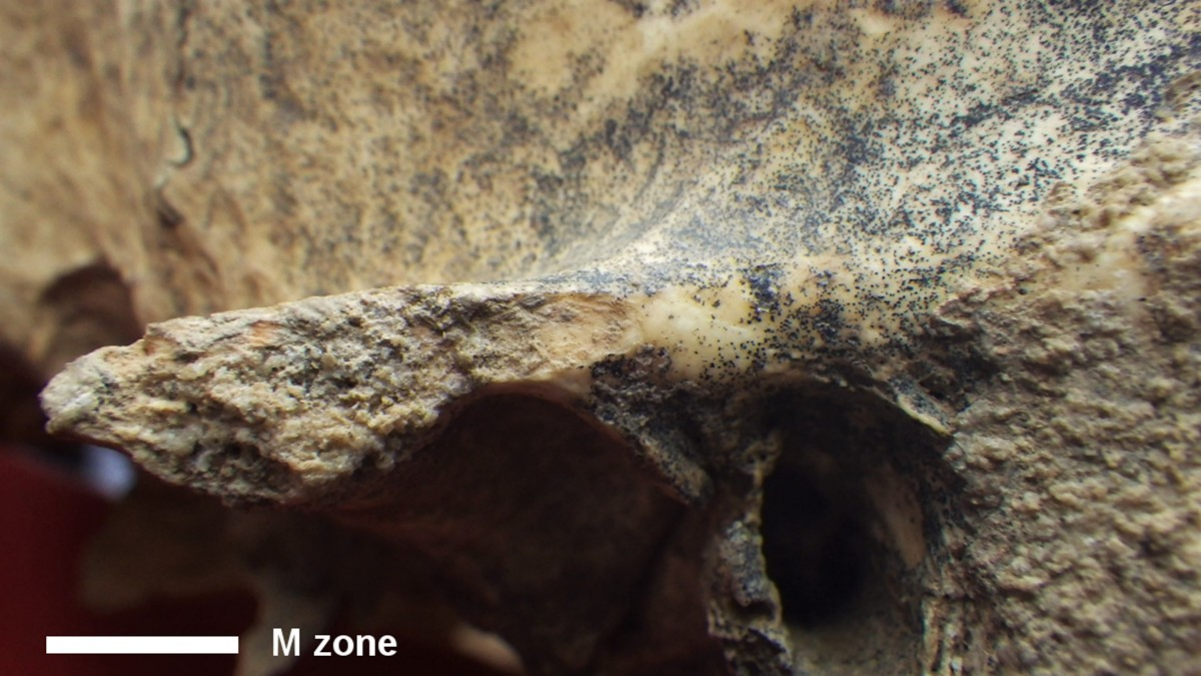
MLC—M Zone on the lateral margin of the left zygomatic process with detail of the superficial detachment of a bone flake.

Various lesions are visible (in the area free from incrustations) on the ectocranial surfaces of the frontal, right parietal, occipital, and left temporal bones. They are extensively described in Table 2 and Figs 10–15.

**Table 2:** Description of the cranial lesions in the frontal (A-G Zones), right parietal (H Zone), occipital (I-L Zones) and temporal (M Zone) bones.

Bone	Position	Description	Interpretation	Note
**Frontal**				
A Zone	Left frontal squama	Seven linear and narrow grooves. The longest one (24 mm) is oriented transversally to the mid-sagittal plane (28 mm far from the left temporal line, 50 mm from the coronal suture). Two other linear grooves are almost parallel to the first one, only 1 mm far from each other. The groove in the middle is 10 mm long, while the anterior one is 5 mm long. Four shorter (about 3 mm) linear grooves, parallel to each other, are perpendicular to the previous ones. The first three are only 1 mm far from each other, while the fourth one–i.e. the medial one–is 3.5 mm apart. The bottom of the marks shows microstriae inside and the same color of the bone surface. Only the longest track shows detachment of some very thin chips.	Perimortem human intervention during cleaning actions	Fig 10A and 10B
B Zone	Left portion of the frontal bone approximately at 2.5 cm from the temporal line	An oval-shaped, slightly bilobed, depressed area (7x5mm) (BA) is located immediately below the temporal line and is uniformly covered by compact tissue with fine parallel striae and by a diffuse, thin dark pigmentation. Immediately below, there is an irregularly shaped area (BB) (10x5mm), finely scraped and grooved by parallel striae. The bottom shows the same color as the rest of surface.	Perimortem human intervention during cleaning actions	Fig 10C
C Zone	Left superior orbital margin, at approximately half its length	Small, slightly depressed and detached area (5x3mm) covered by black pigmentation and crossed in half by a longitudinal mark (5 mm).	Perimortem human intervention during cleaning actions	Fig 10D
D Zone	Between the frontal and left parietal bones	The lesion is discontinuous, crossing the left coronal still open suture. Then, it shows two separated grooves (a longer and irregular one in the frontal bone and another shorter one in the parietal bone) that lied in the same direction.	Postmortem accidental events	Fig 11A
E Zone	Right frontal bone close to the coronal suture	A long (50mm) linear and superficial groove runs almost parallel to the coronal suture. The bottom is quite lighter-colored than the rest of the cranial surface. Only the most lateral part shows the same color as the surrounding cranial surface and irregular grooves.	Uncertain interpretation	Fig 11B
F Zone	Posteriorly placed to E Zone at about 4 mm from the coronal suture	Three marks shorter and larger than the ‘E Zone’ ones. Calcareous incrustations partially cover two of them.	Uncertain interpretation	Fig 11B
G Zone	Right frontal bone 10 mm above the temporal line	Two contiguous areas are detectable: the posterior better preserved than the anterior one. The posterior area about 30 mm from the coronal suture is ellipsoid (major axis about 12 mm) and relatively depressed. Its posterior border shows irregularity (a sort of bone ‘amass’) in continuity with the rest of the surrounding surface, being covered by compact bone. The bottom shows thin parallel microstriae. A sort of interruption separates this area from the more superficial anterior one. The layer of the compact bone of this area is erased, leaving an irregular, abraded, rough and lighter-in-color surface. Its margins are irregular and show the detachment of some superficial flakes. The red color stains almost the entire area of ‘G Zone’.	Antemortem traumatic event (surgery?) with postmortem damage	Fig 12A and 12B
**Right parietal**				
H Zone	4 mm far from the coronal suture and approximately 10 mm above the temporal line	Zone characterized by various irregular pits affecting the compact layer. Two areas with smooth and rounded margins are detectable: the posterior larger (about 5 mm) that the anterior one. Some pits are filled with sediment.	Percussion pits due to perimortem human intervention	Fig 13
**Occipital**				
I Zone	Left border of the fracture in correspondence of the inferior nuchal line, at about 2 cm far from the mid-sagittal plane	Triangular-shaped (about 1 cm height) and peeled area with the same color as the rest of the surface. The central part of this area is stained with little dots of black pigments.	Post- or perimortem events. The black pigmentation is due to manganese oxide	Fig 14
L Zone	On the occipital external protuberance (inion)	A peeled oblong (20x10mm) irregular and rough surface area similar to ‘I Zone’. The color is the same as the rest of the ectocranial surface. Little black dots of pigmentation affect this area and are abruptly interrupted along the left inferior margin of the surface. In the right part of the area, clay sediments fill the irregularities of the surface.		The whole border of the occipital fracture has a curved profile and a beveled irregular margin; its left side is rougher than the right one, with little and larger detached fragments of both sides.
**Left Temporal**				
M Zone	Lateral margin of the left zygomatic process	Superficial detachment of a thin bone flake, resulting in a flat area with a well-defined border (6x3mm) partially spared by sediments. On its superior and inferior margins, an interruption of the dark pigmentation is visible.	Perimortem human intervention (?) and postmortem damage	Fig 15

## Discussion

The discussion will deal with the biological features of the specimen and the interpretation of the numerous lesions, in relation to their time of occurrence (peri-, post-, and antemortem) and possible meanings. The analysis of the sediments and geological context will then be discussed to interpret the features of the deposit and the possible funerary context.

### Biological features

The MLC may be attributed to a 24-to-35-year-old female. Even though only the cranium is preserved, we can provide some general information on the individual’s state of health. As a matter of fact, the evident, generalized porosity in the cranial vault has been described in the literature as being related to an inflammatory bone response [37,38,40]. The traditional interpretation of porotic hyperostosis identifies chronic anaemia (iron or vitamin B_12_ deficiency), likely due to nutritional stress and/or pathogens [55–57]. In palaeopathological records, porotic hyperostosis significantly increased from the Neolithic onward as a result of both the diet, based on agricultural products, and new living conditions in more densely populated settlements, where hygienic conditions were poor and parasites and pathogens were common [58,59]. Two solitary and well-defined mounds of compact bone with a hard, dense, ivory-like appearance (osteomas), observed in the frontal and parietal bones (Fig 9A and 9B), are quite common and are considered benign tumours [37,38,60]. The linear enamel hypoplasia that affects the premolars indicates a disturbance in the normal process of enamel apposition [36]. Considering that P3 and P4 crown formation begins at 4–5 years of age and ends at around 6–7 years [61], it is possible to assume that the individual was affected by prolonged metabolic stress during her childhood. Among dental diseases, caries affected many molars on both sides (LM^1^, LM^3^, RM1, RM^2^, RM^3^), showing a rather high frequency (45%: 5/11), and in one case (RM^2^) the crown was destroyed. Carious lesions began to increase mainly from the Neolithic onward, likely indicating an increased consumption of carbohydrates by farmers [62,63]. This may suggest a diet rich in carbohydrates for the individual examined. Other pathological and physiological conditions, however, may cause this dental condition, such as endocrine disorders, occurring particularly during pregnancy in female individuals [64].

### The interpretation of the cranium lesions

The interpretation of the injuries and the composition of the sediment covering and filling the skull made it possible for us to hypothesize some potential scenarios. The lesions observed in the cranial vault may be due to intentional human intervention, before and around death, on the young female corpse (at least her head) and non-human taphonomic processes occurred after death. None of the lesions observed are attributable to animal actions involving teeth (gnawing), nails, and claws (trampling, scratching) ([44]). In the discussion below, the first comments concern the lesions interpreted as perimortem, with some uncertainty for some of them, followed by those on the postmortem and antemortem lesions.

#### Perimortem and postmortem lesions

The shape, type, and absence of bone repair of the lesions on the left portion of the frontal squama (A Zone) (Figs 2, 10A and 10B) may indicate a perimortem intervention. The groove pattern, the presence of microstriae at the bottom of the grooves, and a colour that is the same as the remaining surface seem to indicate true cut marks (Fig 10A and 10B), likely produced in an attempt to remove soft tissues. This interpretation might be reinforced by the lesions in the B and C Zones situated on the left side of the skull (temporal and orbital margin, respectively) (Figs 2, 10C and 10D). The bone features and colour and the presence of manganese oxide inside both of them indicate old lesions.

Thus it is possible to think that all lesions situated in the left frontal bone may have been produced in an attempt to remove the soft tissues. The meaning of cut marks as a result of practices for the intentional removal of soft tissues from bones has been extensively described [43,65–68]. The cranial vault is covered by a large and dense superficial strip of connective tissue (*galea aponeurotica*) that connects the occipitofrontalis muscle and is attached anteriorly to the frontalis muscle, external occipital protuberance, and highest nuchal lines. The B Zone (Fig 10C) shows a smooth surface and the borders of a BA lesion, likely due to the detachment of a thin bone chip (which likely floated away later due to the action of water), and scrape marks (BB area) could be the result of the detachment of the *galea aponeurotica* and temporal muscle. This muscle originates by a strong aponeurosis from the temporal lines and attaches to the coronoid process of the mandibular bone. The intervention and severing of those tissues may have also caused the disarticulation of the lower jaw, which–likely enlarging the cranial basis–facilitated the access to the brain, as already observed [69]. The lesion of the left orbital border (C Zone) (Fig 10D) could be related to these operations. Quite similar lesions have been observed in other prehistoric samples in a funerary context [70] and in modern samples from defleshing done to prepare a skeleton for teaching purposes (unpublished data).

The lesion on the left zygomatic process (M Zone) (Figs 2 and 15) could not be clearly interpreted. The feature might suggest postmortem damage (see the interruption of the dark pigmentation; Table 2). We could not, however, rule out its interpretation as being related to perimortem interventions, as a possible outcome of actions connected with the disarticulation of the mandible, in particular when excising the masseter muscle connecting the zygomatic process to the external mandibular angle.

The H Zone in the right parietal shows pits probably produced using a sort of tip perpendicularly to the cranial surface (percussion pits) (Figs 2 and 13) ([44]). These lesions seem to be produced perimortem using a tool as a hammerstone or during interventions on the cranial basis placing the skull with the cranial vault on a rough and irregular surface (e.g. stone or gravelly ground).

The long and short marks on the posterior part of the right frontal bone squama (E and F Zones) are of uncertain origin (Figs 2 and 11B). The most lateral part of the long mark (E Zone) shows signs of perimortem lesions, because it seems marked by many passages of a blade, as in a sawing motion. However, the lighter colour of the bottom of the medial part of the E Zone and of the F Zone might indicate that they were caused by accidental postmortem, but not recent, events, taking into account that the F Zone is partly covered by calcareous incrustations. Also, the interruption of the black pigments in the D Zone (Fig 11A) indicates that the mark was caused by postmortem accidental events.

It is likely that the occipital bone lesions occurred postmortem. Calcareous deposits are interrupted quite abruptly along the margins, especially of the L Zone (Fig 14), possibly due to a strong impact that caused the removal of the protruding (the occipital external eminence) parts of the bone. The surface is rough but quite blunt, with well rounded edges, indicating that the postmortem impact likely occurred when the bone was still fresh. The impact may be due to accidental events, although we could not rule out the possibility of intentional perimortem interventions. As regards the large cranial basis lacuna, its rather regular form and the blunt and detached border of the fracture strongly suggest an intentional enlargement of the base. The mandible disarticulation suggested by the lesion on the zygomatic process (M Zone) (Figs 2 and 15) could be related to that action, maybe in order to facilitate the access to the brain. The peeling on the I and L Zones (Fig 14) is similar to that described on animal and human ribs as the distinct result of butchering [43,71–73].

#### Antemortem lesions

The depressed area in the right frontal squama (G Zone) seems to be the result of an antemortem traumatic event. Remodelling processes are visible at the border of the lesion and the presence of thin, parallel grooves on its bottom may be the result of the use of some tools used with force and directed tangentially against the cranial vault for unknown reasons (surgery?) (Figs 2 and 12).

Intentional interventions, sometimes interpreted as therapeutic operations, such as cranial trephination, are well known in the Neolithic and later periods [74]. The non-decorative carbon tattoos of the 5,300-year-old Similaun Iceman (Ötzi), roughly coeval to the MLC, have been interpreted as “medicinal” tattoos to treat chronic or persistent pain [75]. The superficially erased anterior part seems damaged by a more recent postmortem event that left a rough surface, and interrupts the red-coloured area that has been chemically examined to clarify its nature and to test the hypothesis that it may be due to the use of ochre pigment. The chemical analyses confirmed this hypothesis. It is possible that ochre or similar inorganic material was applied around the injury for therapeutic reasons. The possible presence of ochre might be interesting, considering its functional use and numerous (healing, antiseptic) properties, already known from Palaeolithic times, while not ruling out its symbolic meanings, ethnographically documented and hypothesized in archaeological contexts [75–82]. The use of ochre is also attested in a cave (‘Re Tiberio’ Cave, Ravenna) not far from the Marcel Loubens Cave, where anthropic traces extended from the Eneolithic to Iron age [15].

### Thanatocoenosis of the deposit

Regarding the black pigments and calcite crusts, the black Mn coating and the successive carbonate crusts confirm the thanatocoenosis of the deposit: the cranium was brought into the cave by a small creek at a time when the shaft below was not yet formed, and successively embedded in loamy sediments during a period when the sinkhole was becoming clogged and was then progressively abandoned. The by the active water flow. The Mn coating was formed inside the sediment by reduced Mn-rich waters that were slowly oxidized. Only later, when the sinkhole was reactivated again, new CO_2_-rich infiltration waters dissolved the gypsum and caused the formation of new flowstones and carbonate crusts on the cranium. In this warmer and wetter period, the 12-m shaft was formed, and the cranium remained suspended high above the shaft’s floor until its discovery.

In gypsum areas, and especially in large dolines, sinking points tend to be stable (and active) over short periods of time (typically less than a century), with the evolution of the doline surface forcing waters to find alternative sinking points upstream of the active ones. Once such a sinking point is abandoned, it fills up with debris and can stay closed for long periods, until favourable conditions can reactivate these passages once again.

**Fig 16:**
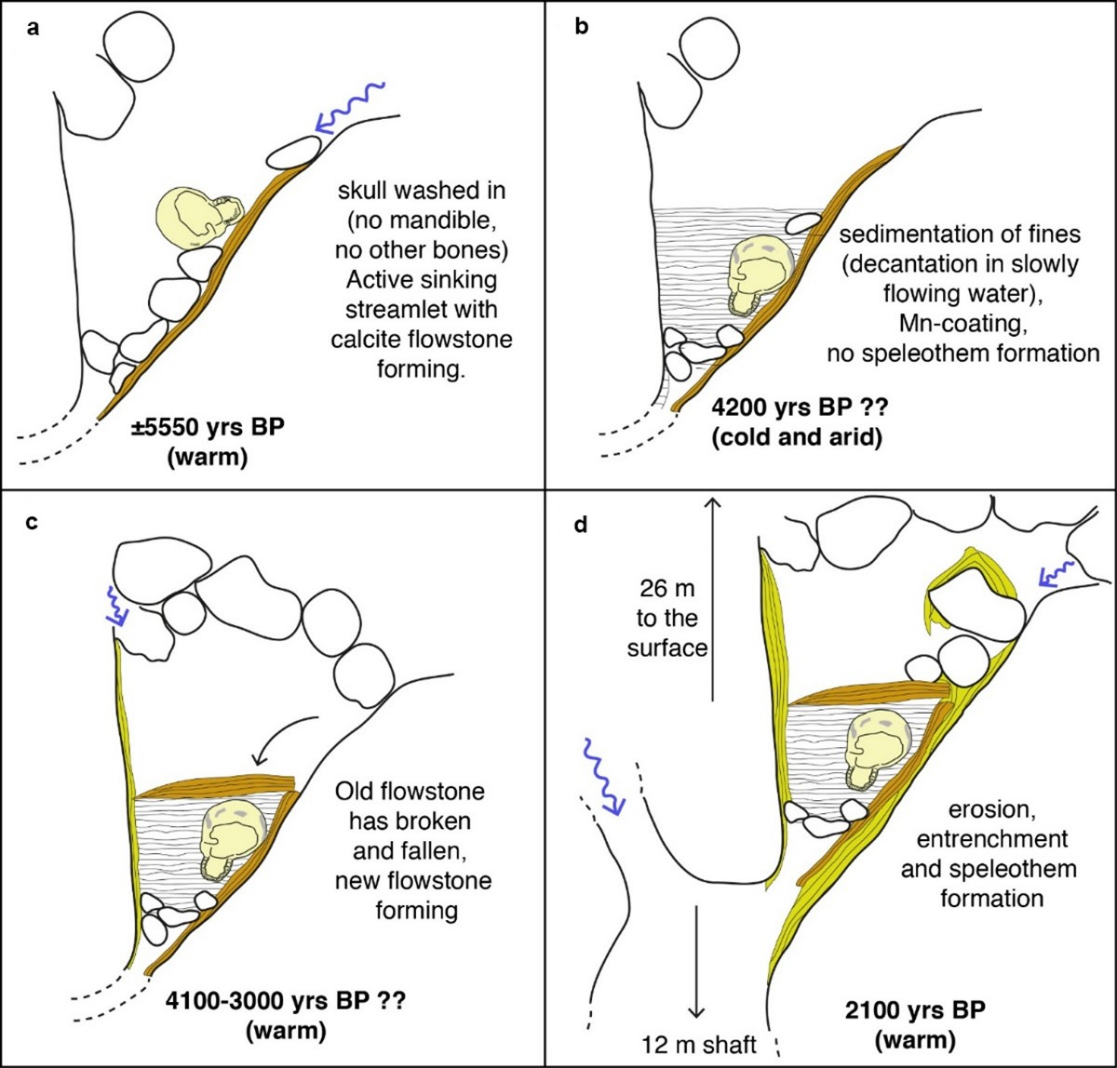
Reconstruction of the sequence of the taphonomic events: MLC enters the doline (a), initial deposition of sediments (b), flowstone is added to the deposition (c), and erosive and entrenchment processes occurred (d).

The cranium was vertically situated, exposing its basilar view (cranial basis, palate, and occipital bone) and was embedded in a loamy matrix, representing a mudflow in a low-gradient environment (Fig 16A and 16B). These sediments, including the cranium, rested on an old flowstone formed while the sinkhole was still active, before the sediments were deposited in the cave, probably due to the progressive occlusion (and abandonment) of the sinking point. After the sedimentation stopped, erosion again began to destabilize part of the old flowstone, a slab of which collapsed onto the sediment containing the cranium, thus protecting it from further erosion and transport (Fig 16C). These newly infiltrating waters, rich in CO_2_, were able to deposit a new flowstone generation, partly eroding the sediment in contact with the walls. The reactivated cave passage started evolving downward, with the formation of a lateral sinking creek and carving out the maze lying below. This new reactivation was able to entrench approximately 12 metres of gypsum, connecting to the lowering base level (Fig 16D).

The high-frequency alternation of cave formation-sedimentation periods has been documented in many gypsum caves in the northern Apennines, and are often climate-driven [83]. The final entrenchment responsible for the 12-metre-deep shaft creation, likely developed in a warm and wet period (2100 yrs. BP?), caused the cranium and the sediment to remain suspended high above.

This reconstruction matches the observations on sediments in the cranial cavity. The type of material filling the cranium cavity, i.e. sandy pelite with mm-sized angular debris, micro wood charcoals, gypsum, and a few gastropod shells typical of calcareous substrata, suggest that the cranium was filled during transport toward its final resting place. The recovered sedimentary and fossiliferous features also indicate debris flow (or torrent flow) as a possible transport carrier. Indeed, such water-saturated processes are also common after intense wildfires, as combustion particles make the ground surface hydrophobic and facilitate the formation of a drainage network by rainwater across the burnt area, originating small debris flows that contribute to draining water-saturated masses of sediment to streams or sinkholes.

Thus it is possible to hypothesize that the body (or parts of it) was situated (likely exposed?) at the edge of a doline–probably at an advanced skeletonized stage, but still retaining some fresh osseous tissue–and, due to movements of the terrain, moved from its original position, becoming disarticulated and dismembered. Meanwhile, the skull might have rolled away, generating the postmortem injuries to the occipital bone (which seem to have been produced on a fresh osseous tissue) and perhaps the large parietal fracture, as it was likely drawn by the water, debris, and mudflow. It is possible to suppose that if the skeleton was intact by the time of this sequence of events, other skeletal elements, different in shape and size, might have remained stuck elsewhere and dispersed during transportation. The cranium would have rolled more easily than other skeletal parts in a water stream and debris flow (cf. [84]), already in absence of the mandible (perhaps actively removed after death), since both glenoid fossae, especially on the left side, show the same dark pigmentation as the rest of the cranium, before arriving at the cave. During its decomposition and those dynamic phases, it would have been filled with sediment. Therefore, it would have reached the cave and come to a stop on the plateau where it was found.

### The funerary context

The isolated MLC, even though it probably fell by accident into the cave where it was found, a place devoid of any archaeological finds, offers important insights into the peri- and/or postmortem practices of the Eneolithic populations of northern Italy. As far as we know, this is the first Italian Eneolithic cranium where evidence of intentional perimortem human intervention has been found. The findings and secondary deposition of crania in caves are well documented, but we were unable to find any reports of peri- or postmortem lesions.

In Eneolithic funerary contexts in Italy, scattered and commingled bones have been interpreted as the result of intentional disturbance and manipulation practices. For example, skeletal manipulation with a particular predilection for crania which are displaced within the same tomb chambers in niches or different tombs, are well known within the so-called Rinaldone Culture, widespread in the Tuscany, Northern Latium, and Marche regions [85–88].

In northern-central Italy, two main rituals have been recognized, corresponding to different geographical-ecological environments: simple pit graves in necropolis contexts in the central-eastern Po Valley, and collective burials in the Alpine region and Apennines, especially inside caves or under rock shelters. However, this distinction is not always so clear because, in some cases, the two above-mentioned funerary rituals coexist, as in Lombardy, Veneto and Emilia Romagna [19,89–93].

The use of caves, natural cavities, and rock shelters as places of collective burials is an Eneolithic period phenomenon that has been well known since the 19^th^ century, thanks to the explorations of Father Gaetano Chierici, who discovered the presence of human remains belonging to male and female adults and subadults in the ‘Tana della Mussina’ Cave (Reggio Emilia, Emilia Romagna). Some of the skeletons there appeared disarticulated, others partially burnt, and they were considered part of a collective burial site that was frequented at various times [14,94]. These rituals have been interpreted as aimed at reinforcing social cohesion and collective memory throughout the cult of ancestors [15,17,18,86]. They have been observed in both the Apennine area and the Alpine and pre-Alpine region: Lombardy [91], Veneto [18,92], Trentino [95] Northern Tuscany [96], and Liguria [97]. In the Lombard pre-Alpine region, the funerary practices documented at ‘Riparo Valtenesi di Manerba’ (Brescia) [98] suggest the existence of distinct, specific areas, intended for different operations on human remains: a place where the decomposing bodies were kept for the subsequent manipulation of their remains and another place used as a proper ossuary, where bones were transferred for the performance of new rituals. This hypothesis has been confirmed in other caves and rock shelters of the pre-Alpine arc [91], and the custom may be considered part of a widespread cultural phenomenon. In Liguria, in the ‘Da Prima Ciappa’ Cave in Val Frascarese (Genoa) [99], a calotte and a calvarium were found facing the entrance and resting on a mound of long bones.

In Emilia Romagna, numerous caves have been systematically explored by speleologists and archaeologists since the 19^th^ century. Anthropic presence and funerary contexts are well documented in a period between the Eneolithic and the dawn of the Bronze Age [15,16]. In the province of Bologna (the same area where the Marcel Loubens Cave is located) human remains have been found in the ‘Sottoroccia del Farneto’ (only 600 m away from the Marcel Loubens Cave, Eneolithic [7,100]), in the Farneto Cave (Early Bronze Age 2; [13]), and in the ‘Grotta di fianco alla chiesa della Gaibola’ (Eneolithic, Early Bronze Age; [7]).

In the ‘Sottoroccia del Farneto’ many commingled and highly fragmented human remains were discovered in the 1960s [11,12] and, in part, studied to reconstruct their biological profiles [8–10]. However, the lack of archaeological documentation regarding the removal of human remains, typical for those years, means the context remains unclear. The anthropological re-examination performed to distinguish the non-human taphonomic vs the anthropogenic origin of the highly fragmented bones has revealed the presence of perimortem lesions related to an intentional treatment of the cadaver [101,102].

A few kilometres away from the Marcel Loubens cave, in the 1930s a human male calotte (now lost) [103] was discovered in a shaft near the entrance of the Gaibola Cave (‘Grotta di fianco alla chiesa della Gaibola’, Bologna) together with bear and wild boar teeth, flint flakes, and pottery fragments [104]. In the same cave, in a chamber named ‘Sala dello Scheletro’ (Skeleton Chamber), many other human bones were found in a layer containing pottery fragments, charcoal particles, stone tools, and red ochre. Under this layer, an incomplete male skeleton (lacking the cranium, while the mandible remained *in situ*) lying on its right side in a flexed position, surrounded by abundant clots of red ochre, was found together with some pierced animal teeth and shells. The lack of some bones has been attributed to the action of a small water stream that formed after the burial took place. Archaeological materials suggested that the first layer belonged to the final Eneolithic [7]. However, the presence of the skeleton with missing cranium could suggest the presence of burials inside caves from where crania were easily removed, accidentally (by water?) or even intentionally. Whether or not the cranium in the shaft belonged to the incomplete male skeleton is impossible to ascertain, but cannot be ruled out. This could suggest a scenario that can apply to the MLC find, also.

Human skeletal remains were also documented in caves in the area of ‘Gessi Romagnoli’ (Ravenna): ‘Re Tiberio’ Cave (Eneolithic and Early Bronze Age 1; [15,105]), ‘Tanaccia di Brisighella’ and ‘Banditi’ caves (Eneolithic/Early Bronze Age?; [16,105]). The ‘Re Tiberio’ Cave contained the remains of 16–17 individuals, apparently without a selection based on sex or age. Some skeletal parts were in primary deposition, but most of the bones were commingled and displaced. In some places inside the cave, long bones were arranged in an apparent order, indicating secondary interventions. The almost complete absence of crania leads to the same conclusion, especially considering that some maxillary teeth were found [15,105]. In the Tanaccia cave, two crania (one of an infant and one of an adolescent) were placed in a niche together with a vase and a pottery fragment, while in the ‘Banditi cave’ the mandible of a 6-year-old child was found in a hearth ([105]).

In Emilia Romagna, in addition to the above-mentioned funerary contexts in natural cavities, large necropolises with simple pit graves are well documented, such as the most famous ones in Spilamberto (Modena) [106–108] and ‘Celletta dei Passeri’ (Forlì-Cesena), where manipulation, removal and postmortem intervention on skeletal remains are documented [109]. The recent anthropological review of some previously studied Eneolithic skeletons of the open site of ‘Fornace Cappuccini’ (Ravenna)–where burials have been found in the artificial trench around the residential area [110,111]–has provided some evidence of perimortem treatment on a cranium, in particular scrape marks located at the insertion of the temporal muscle on the right parietal bone [112].

Even in earlier times, peri- and postmortem treatments of human remains, especially skulls, in funerary contexts are largely documented. Here we just recall two Southern Italy Neolithic sites (Scaloria Cave and ‘Passo di Corvo’, both in Puglia) recently studied from an anthropological and taphonomic point of view that allowed to detect perimortem treatment of the corpses, offering new evidence regarding the funerary behaviour of those peoples [113–117]. In Emilia Romagna, during the Neolithic Culture VBQ, cranial manipulations are attested within some funerary contexts as postmortem activities [118]. We may suppose that the habit of manipulating the skulls might have been transmitted to the Eneolithic culture, where, as we have shown in the study of the specimen MLC, also the perimortem manipulation is clearly attested. Thus, to better understand the mortuary behaviour of Neolithic and Eneolithic peoples, an anthropological and taphonomic review of the previously studied human remains is in need.

## Concluding remarks

The discovery of the cranium in the Marcel Loubens Cave dated to the early phase of the Eneolithic (between 3,600 and 3,300 B.C.) provides new insights for the interpretation of the anthropic presence in northern Italian areas and, more in general, throughout the Italian territory, adding new data about the perimortem treatment of corpses, possibly in a funerary context.

Our results suggest that the body of a young woman (or her head/cranium) was situated at the edge of a doline, most likely coming from an unknown burial place or other type of ritual context. Actually, the presence of cranial perimortem injuries and the comparison with some other archaeological contexts enable us to hypothesize that the body of this young woman was subject to treatment and manipulation within a funerary or other ritual context. Postmortem traumas indicate that the cranium (intentionally or accidentally removed from the body) rolled away and fell accidentally into the shaft of the Marcel Loubens Cave, where it was preserved and later found. Finally, the possible relationship between the antemortem cranial injury, likely treated with ochre, and the after death destiny of the woman remains completely unknown.

It is noteworthy that anthropological and taphonomic analyses, together with geological and speleological observations, made it possible for us to shed light on that context in the absence of any archaeological evidence. The presence of perimortem and postmortem injuries has contributed to the reconstruction of the manner and timing of the manipulation of the body and head, likely for the purpose of removing the soft tissues and lower jaw (likely to facilitate the access to the brain), up to the occurrence of the accidental natural events that brought the specimen to the shaft of the cave. We do not know if the context referred to a formal burial or other ritual context.

## Supporting information

S1 Table Cranial measurements taken from Howells (1989) and recorded from the modern identified human skeletal collection from Certosa cemetery of Bologna (Italy) are abbreviated as follows: GOL, maximum cranial length; NOL, nasio-occipital length; XCB, maximum cranial breadth; XFB, maximum frontal breadth; STB, bistephanic breadth; AUB, biauricular breadth; WCB, minimum cranial breadth; ASB, biasterionic breadth; NPH, upper facial height; NLH, nasal height; MAB, maxillo-alveolar breadth; MDH, mastoid height; MDB, mastoid breadth; ZMB, bimaxillary breadth; WMH, cheek height; GLS, glabella projection; FRC, frontal chord; FRS, nasion-bregma subtense; FRF, nasion subtense fraction; PAC, parietal chord. *Stepwise selected measurements.
(XLSX)

S1 Video MLC—Video of the sequence of the slices of the cranium (first CT scan). (AVI)

S2 Video MLC—Video of the of the slices of the cranium (second CT scan). (AVI)

S3 Video MLC—Video of the sequence of slices of the maxilla (CT scan). (AVI)
